# Organohalide Respiring Bacteria and Reductive Dehalogenases: Key Tools in Organohalide Bioremediation

**DOI:** 10.3389/fmicb.2016.00249

**Published:** 2016-03-01

**Authors:** Bat-Erdene Jugder, Haluk Ertan, Susanne Bohl, Matthew Lee, Christopher P. Marquis, Michael Manefield

**Affiliations:** ^1^School of Biotechnology and Biomolecular Sciences, University of New South WalesSydney, NSW, Australia; ^2^Department of Molecular Biology and Genetics, Istanbul UniversityIstanbul, Turkey; ^3^Department of Biotechnology, Mannheim University of Applied SciencesMannheim, Germany

**Keywords:** reductive dehalogenase, organohalide respiration, bioremediation, *Dehalobacter*, *Dehalococcoides*

## Abstract

Organohalides are recalcitrant pollutants that have been responsible for substantial contamination of soils and groundwater. Organohalide-respiring bacteria (ORB) provide a potential solution to remediate contaminated sites, through their ability to use organohalides as terminal electron acceptors to yield energy for growth (i.e., organohalide respiration). Ideally, this process results in non- or lesser-halogenated compounds that are mostly less toxic to the environment or more easily degraded. At the heart of these processes are reductive dehalogenases (RDases), which are membrane bound enzymes coupled with other components that facilitate dehalogenation of organohalides to generate cellular energy. This review focuses on RDases, concentrating on those which have been purified (partially or wholly) and functionally characterized. Further, the paper reviews the major bacteria involved in organohalide breakdown and the evidence for microbial evolution of RDases. Finally, the capacity for using ORB in a bioremediation and bioaugmentation capacity are discussed.

## Chlorinated substances as environmental pollutants

Organohalides are recalcitrant environmental pollutants contaminating soil and groundwater. Across the atmosphere, pedosphere and the oceans there are over 2000 recognized chlorinated organic natural products with over 1500 produced biologically and the remainder formed through abiotic processes (Gribble, 2003, 2010). Organohalide contamination of land and water which is of anthropogenic origin (e.g., arising from the manufacture and use of pesticides, dry cleaning solvents, ozone-depleting refrigerants, industrial degreasers) is widespread and poses significant potential danger related to their adverse impacts on health and effects on ecosystems. Among the commonly reported anthropogenic organohalides are hexachlorobenzene (HCB), trichloromethane (TCM), polychlorinated biphenyls (PCBs), perchloroethene (PCE), trichloroethene (TCE), trichloroethanes (TCA), dichloroethanes (DCA), polybrominated diphenyl ethers (PBDEs), chlorinated/brominated phenols, and dioxins. Pollution of terrestrial and aquatic systems caused by the excessive release of the anthropogenic organohalides, has led researchers to search for strategies to remediate contaminated sites using microbiota which includes a diverse range of mostly anaerobic bacteria.

**Figure 1:**
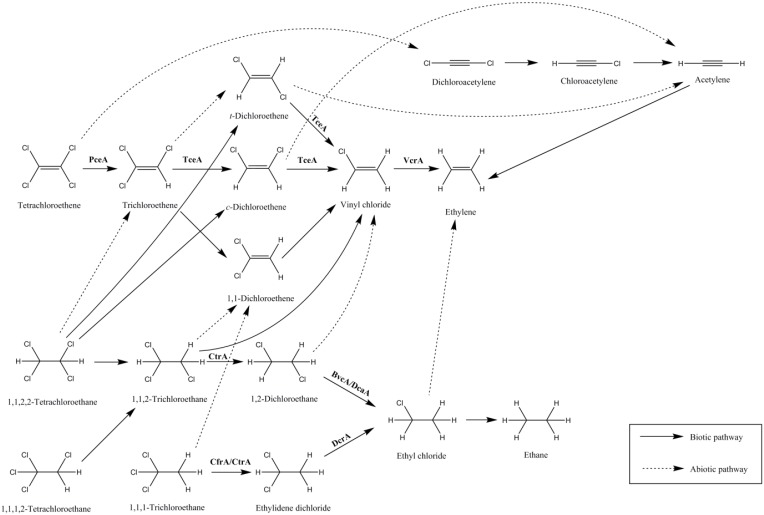
**Dechlorination of chlorinated ethenes and ethanes via anaerobic biotic and abiotic pathways**. Examples of enzymes catalyzing the biotic reactions are given: PceA (Miller et al., 1998), TceA (Magnuson et al., 2000; Fung et al., 2007), VcrA (Parthasarathy et al., 2015), CtrA (Zhao et al., 2015), BvcA (Tang et al., 2013), DcaA (Marzorati et al., 2007), CfrA (Tang and Edwards, 2013), CtrA (Ding et al., 2014), and DcrA (Tang and Edwards, 2013).

Polychlorinated ethenes and ethanes are major members of organohalide pollutants and their degradation can occur via anaerobic biotic and abiotic pathways (Figure 1). Chlorinated ethenes, mainly PCE and TCE, used as a dry-cleaning solvent and an industrial degreaser respectively, are among the most abundant pollutants. The products of dechlorination of PCE and TCE are *cis*-dichloroethene (*cis*-DCE), vinyl chloride (VC), and ethene, and many ORB have been found to catalyze their dechlorination partially or completely (Middeldorp et al., 1999). However, only a limited number of RDases that can dechlorinate PCE (PceA), TCE (TceA), and VC (VcrA) have been biochemically characterized (Table 1). Another group of organohalide pollutants are chlorinated ethanes, such as tetrachloroethane (TeCA), trichloroethane (TCA), and dichloroethane (DCA). In particular, 1,1,1-TCA (methyl chloroform), used as a solvent and in many consumer products, is one of the most predominant environmental pollutants and has the potential to cause serious health issues (Padilla-Crespo et al., 2014). 1,1-DCA, 1,2-DCA, and VC are also common groundwater contaminants and are classified by the US Environmental Protection Agency as a possible human carcinogen (Group C), a probable human carcinogen (Group B2) and human carcinogen (Group A), respectively (accessed Dec 2015; http://www.epa.gov/). Chlorinated methane pollutants, for example, trichloromethane (TCM), are highly hydrophobic and volatile. They are manufactured as industrial solvents for organic materials and also as intermediates for the production of polytetrafluoroethylene and the refrigerant monochlorodifluoromethane (HCFC-22; Justicia-Leon et al., 2014). Due to the poor solubility of TCM in water (<8 g/L, 20°C), high density (ρ = 1.48 g/cm^3^), and long half-life (3500 years), this highly recalcitrant organohalide sinks and stays as solvent pools within subsurface water systems, prompting urgent, and efficient remediation measures (Mabey and Mill, 1978; Lee et al., 2015). Fortunately, some ORB have been identified that can utilize these organohalides as terminal electron acceptors, and some RDases responsible for their reductive dehalogenation have been reported.

**Table 1:** **Biochemical properties of reductive dehalogenases purified in native form**.

**Enzyme**	**Organism**	**Methods**	**Reaction catalyzed**	**MW (kDa)^a^**	**Specific activity, (μmol/min/mg)^b^**	**References**
TeCH RDase	*Flavobacterium* sp	Protamine sulfate treatment → Ammonium sulfate fractionation → Phenyl-agarose → DEAE-agarose → Mono Q → Gel filtration	Tetrachloro-*p*-hydroquinone (TeCH) → TCH & DCH	30	0.123	Xun et al., 1992
3Cl-BA-RDase	*Desulfomonile tiedjei* DCB-1	Protamine sulfate treatment → Ammonium sulfate fractionation → Membrane solubilization (CHAPS or Triton X-100) → High Q → Hydroxyapatite → Octyl agarose	3Cl-BA → Benzoate	64 and 37	0.019	Ni et al., 1995
PceA	Mixed enrichment culture of *Dehalococcoides mccartyi* 195 (formerly *D. ethenogenesis* 195)	Ultracentrifugation → Membrane solubilization (Triton X-100) → POROS HP/M → POROS PH/M	PCE → TCE	51	20 (TCE)	Magnuson et al., 1998, 2000
TceA	*Dehalococcoides mccartyi* 195 (formerly *D. ethenogenesis* 195)		TCE → Ethene 1,2-DCA and 1,2-DBA to ethene	61	12.1 (VC) 7.5 (1,2-DCA)	
VcrA	*Dehalococcoides mccartyi* sp. VS	Ultracentrifugation → Membrane solubilization (CHAPS) → HighTrap Q → Superose 6	VC and All DCE isomers → Ethene	62	1.0 (VC)	Müller et al., 2004
PceA	*Sulfurospirillum multivorans* (formerly *Dehalospirillum multivorans*)	Ultracentrifugation → Q-Sepharose HP → Ammonium sulfate precipitation → Phenyl-Superose HR → Superdex™ 75-pg	PCE → TCE → *cis*-1.2-DCE	57	158	Neumann et al., 1996
CprA	*Desulfitobacterium dehalogenans*	Ultracentrifugation and Membrane solubilisation (Triton X-100) → Q-Sepharose → Mono Q (pH 6.0) → Mono Q (pH 7.8)	Dechlorination of various ortho-chlorophenols (i.e., Cl-OHPA and 2, 3-DCP)	48	28	van de Pas et al., 1999
CprA	*Desulfitobacterium* sp. PCE-1	Membrane fractionation and solubilization, Q-Sepharose, MonoQ pH 6.5 & 7.8	Dechlorination of 3-chloro-4- hydroxyphenylacetate		4.09	van de Pas et al., 2001
PceA			PCE → TCE		5.51	
PceA	*Desulfitobacterium hafniense* TCE-1	Membrane fractionation and solubilization, MonoQ pH 6.5 & 7.8	PCE → TCE → *cis*-DCE		10.0	van de Pas et al., 2001
CrdA	*Desulfitobacterium hafniense* PCP-1 (formerly *D. frappieri*)	Ultracentrifugation → Ammonium sulfate precipitation → methyl HIC → Ammonium sulfate precipitation → Protein Pak 300 SW	2,4,6-TCP → 2,4-DCP	33.8	0.38	Boyer et al., 2003
CprA5	*Desulfitobacterium hafniense* PCP-1 (formerly *D. frappieri*)	Membrane solubilization → Protein Pak DEAE-5PW → Methyl-HIC	3,5-DCP → 3-CP	57	0.4	Thibodeau et al., 2004
PceA	*Desulfitobacterium hafniense* Y51	Hydroxyapatite → butyl-Toyopearl 650 M → Chromatofocusing	PCE → TCE → *cis*-1,2-DCE	58	0.11	Suyama et al., 2002
PceA	*Dehalobacter restrictus*	Ultracentrifugation and Membrane solubilization (Triton X-100) → Q-Sepharose	PCE → *cis*-1,2-DCE	60	11.9	Schumacher et al., 1997

a *As reported by authors, using different methods*.

b *One unit of enzyme activity is defined as the reduction of 1 μmol of substrate per min per mg enzyme*.

## Reductive dehalogenases in organohalide respiration

The discovery made three decades ago that certain anaerobic bacteria could derive their energy by reducing organohalides, has motivated an intensive research effort in this field (Shelton and Tiedje, 1984). These ORB are equipped with concerted membrane associated proteins that drive the organohalide respiration process. The ultimate goal of this respiratory process is to synthesize ATP driven by a proton motive force (PMF) established across the cytoplasmic membrane (CM). A chemiosmotic coupling between reductive dehalogenation and ATP synthesis has been proposed, as uncouplers significantly reduce the ATP pool relative to the reductive dechlorination rate (Louie and Mohn, 1999).

The reductive dehalogenation reaction is highly exergonic, as demonstrated for most organohalides with the free energy (Δ*G*°′) of dechlorination with hydrogen as the electron donor in a range of between −131 and −192 kJ/mol. Furthermore, the organohalides are thermodynamically favorable as electron acceptors under anaerobic conditions, as their standard redox potential (*E*°′) lies between approximately +250 and +600 mV (El Fantroussi et al., 1998; Holliger et al., 1998). Although such an energy yield could potentially result in 2.5–2.7 ATP molecules per molecule of chloride ion released, considering 70 kJ is required to make one mole of ATP in a living cell (Schink and Friedrich, 1994), biomass yields per mole of chloride released for ORB are generally low. This possibly stems from the fact that only two mole H^+^ per mole H_2_ oxidized are released and three moles of H^+^ are required to generate sufficient proton-motive force to produce one mole of ATP (Schumacher and Holliger, 1996).

**Figure 2:**
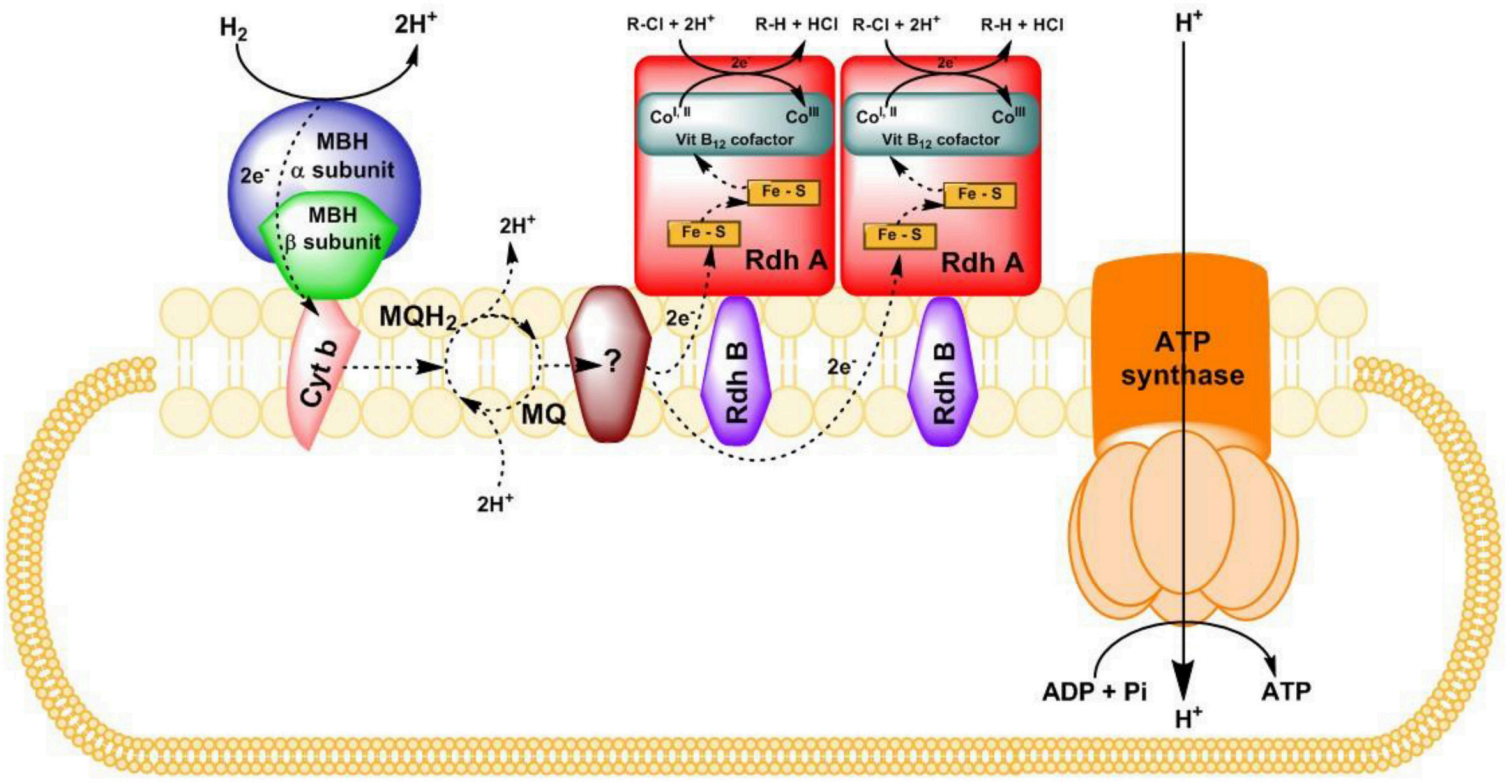
**Putative representation of electron transfer chain with H_**2**_ as electron donor, and organohalide as electron acceptor in ***S. multivorans*** (derived from Bommer et al., 2014 and Goris et al., 2014)**. Rdh A, reductive dehalogenase catalytic subunit; Rdh B, reductive dehalogenase membrane anchor protein; MBH, membrane-bound uptake hydrogenase; Cyt b, cytochrome *b* subunit of the MBH; MQ, menaquinone; MQH_2_, dihydromenaquinone; R-Cl, organohalide.

Reductive dehalogenases are central to this process. The catalytic subunit, reductive dehalogenase homologous A subunit (RdhA) of the enzyme harbors a cobalamin (vitamin B12) cofactor and two Fe-S clusters (Figure 2). It had been debated whether RdhA “faces” the cytoplasm or periplasm, and whether it is monomeric or dimeric. A recent structural study demonstrated attachment to the periplasmic side of the CM in a dimeric form (Bommer et al., 2014). In Figure 2, a putative electron transfer chain is depicted based on the biochemical pathway found in *Sulfurospirillum multivorans* (Bommer et al., 2014; Goris et al., 2014). RdhA is anchored to the CM via its membrane anchor protein (RdhB). Reductive dehalogenation occurs at the catalytic site where reduced cobalamin cofactor (Co^I, II^) attacks the halogen atom of an organohalide to cleave a carbon-halogen bond, leading to sequential removal of the halogen substituents from the organic backbone. The cleavage of the carbon-halogen bond was proposed to occur either heterolytically or homolytically (Jugder et al., 2015b). The electrons required for the reduction of a halogen ion are transferred to the Co ions through distal and proximal Fe-S clusters.

The coupling between oxidation of the electron donor (in most cases, hydrogen or formate) and reductive dechlorination of electron acceptors (organohalides) is believed to drive electron transport phosphorylation, where membrane associated oxidoreductases are involved. Membrane-bound hydrogenases (MBH) are the initial oxidizers to take up the electrons released from molecular hydrogen (Jugder et al., 2013, 2015a). Both uptake (Hyd or Hup type) and energy-conserving hydrogenases (Hyc or Hym type) have been proposed to play a role in organohalide respiration (Morris et al., 2006; Rupakula et al., 2013; Kruse et al., 2015). Elevated expression of periplasmic formate dehydrogenase at transcriptional and translational levels strongly suggested its role in organohalide respiration where formate is used as an electron donor (Kruse et al., 2015). Surprisingly, the same pattern was also observed in *Dehalococcoides* cells where formate cannot be used as the electron donor. In this organism, serine substitution at the critical site was revealed in the formate dehydrogenases found via in-depth phylogenetic analysis (Morris et al., 2006). However, the potential role of the periplasmic formate dehydrogenase in organohalide respiration is unresolved, requiring further biochemical investigations.

For subsequent transfer across the membrane, the electrons are taken up by electron carrier(s), such as menaquinone that could possibly function as proton translocating coenzymes to release the protons on the cytoplasmic side of the membrane during the redox reaction in *Dehalobacter* (Schumacher and Holliger, 1996) and *Desulfitobacterium* (Kruse et al., 2015) species. However, the involvement of menaquinone in electron transfer in *S. multivorans* was questioned due to its high redox potential (−74 mV) resulting in its unlikely role in direct electron delivery to RDases (Miller et al., 1996). Thus, the involvement of additional unknown electron carriers to subsequently generate the PMF has to be envisaged. Recent studies have proposed a putative membrane bound quinol dehydrogenase as a potential candidate linking menaquinone and PceA in *S. multivorans* (Goris et al., 2014) or CprA in *D. dehalogenans* (Kruse et al., 2015). Moreover, an extracellular flavoprotein was postulated to act as an electron shuttle between the quinol dehydrogenase and the CprA (Kruse et al., 2015). Despite these findings, there is no real consensus on the exact mechanism or mechanisms of the cellular respiratory pathway or on the involvement of various membrane associated components.

## Microorganisms producing reductive dehalogenases

Most ORB are strict anaerobes, characterized by slow growth, light and pH sensitivity, and dependency on external supply of corrinoid cofactors. ORB are known to thrive within mutualistic anaerobic microbial communities, rather than in pure culture (Maphosa et al., 2012). Since the isolation of the first ORB, *Desulfmonile tiedjei* (DeWeerd et al., 1990), multiple genomes and metagenomes of ORB have been reported. The average genome size ranges from 2.6 to 3.1 Mb with an average GC content of about 44–45% (Richardson, 2013). Beyond detoxification, ORB are also an integral part of the global biogeochemical chlorine cycle between the oceans and the atmosphere (Krzmarzick et al., 2012).

RDases from *Dehalococcoides, Dehalobacter*, and *Desulfitobacterium* strains are amongst the most extensively studied of these enzymes. Due to their degree of dependency on organohalide respiration, ORB are classified as either obligate organohalide respirers that are highly specialized with very restricted metabolism or non-obligate organohalide respirers that are characterized by their capacity to use a broader range of electron acceptors and donors (Maphosa et al., 2010).

The growth profiles of *Dehalobacter* and *Dehalococcoides*, the main niche specialists from Firmicutes and Chloroflexi, respectively, are strictly dependent on organohalide respiration with mostly polychlorinated benzenes or chlorinated aliphatic hydrocarbons as electron acceptors and H_2_ as the sole electron donor. Although pure cultures of these obligate species can be onerous to cultivate due to their slow growth rate and fastidious nutritional requirements, the cultures used in bioremediation for bioaugmentation are typically mixed enrichment cultures for which nutritional requirements are satisfied with a mineral medium containing typically a fermentable substrate and the chlorinated electron acceptor. There have been several recent studies with enrichment cultures relevant for bioremediation showing progress on growth methods to yield high concentrations of these microbes and doubling times for *D. mccartyi* of 17 h (Vainberg et al., 2009; Delgado et al., 2014). Recently discovered *Dehalobacter* and *Desulfitobacterium* strains have been revealed to use TCM for their reductive respiratory process, and some of their respective RDases have also been reported (Tang et al., 2012; Tang and Edwards, 2013; Ding et al., 2014). In our lab, we have identified a mixed community containing *Dehalobacter* that completely transformed TCM to acetate and hydrogen (Lee et al., 2012), and subsequently a novel TCM-respiring *Dehalobacter* sp. strain UNSWDHB was isolated from the mixed community and genome sequenced (Deshpande et al., 2013). In contrast, *Desulfitobacterium* species are metabolic generalists within the Firmicutes. They are relatively easy to culture with regard to their capacity to utilize a range of electron acceptors, allowing extensive studies on these organisms and their respective enzymes.

## Production, purification, and characterization of RDases

Between the late 1990s and the early 2000s, several efforts to obtain pure native RDases were reported (Table 1). Since RDases are membrane-associated, the use of ultracentrifugation for membrane fractionation followed by different solubilization techniques was commonly used as initial purification steps. Commonly this was followed by chromatographic separation utilizing ion exchange, hydrophobic interaction, and size exclusion matrices. For the most part, this resulted in partially purified proteins with varied specific activity. The PceA from *S. multivorans* (Neumann et al., 1996) represents the highest specific activity reported (158 μmol/min/mg), however, the range of activities reported are broad, with activities <1 μmol/min/mg described. Indeed, due to the difficulties with producing and purifying functional native enzymes, efforts to express these enzymes heterologously have arisen, with recent successes in generating functional enzymes (Jugder et al., 2015b) described (summarized in Table 2). Early efforts to express recombinant PceAs in *E. coli* failed to obtain functionally active enzymes (Neumann et al., 1998; Suyama et al., 2002; Kimoto et al., 2010; Sjuts et al., 2012), however, recently three research teams successfully developed expression and purification strategies to obtain catalytically active RDases in different heterologous hosts (Table 2). Specific activities reported for these enzymes are at the lower end of the range reported for native enzymes (2–10 μmol/min/mg). The demonstrated success with purifying a number of the native enzymes suggests that this approach cannot be ignored for generating enzymes for biochemical and structural characterization, however, early promise shown in recent heterologous expression of RDases will encourage further developments in recombinant expression strategies.

**Table 2:** **Biochemical properties of reductive dehalogenases expressed in recombinant systems**.

**Enzyme**	**Strain**	**Expression host**	**Affinity tags**	**Maturation proteins**	**Specific activity, (μmol/min/mg)**	**Features**	**References**
PceA	*Sulfurospirillum multivorans* (formerly *Dehalospirillum multivorans*)	*E. coli* BL21 (DE3)	NR	NR	catalytically inactive		Neumann et al., 1998
PceA	*D. hafniense* strain Y51	*E. coli* BL21 (DE3)	Trx (thioredoxin protein) Tag, S Tag, and His Tag at the 5′ end and His Tag at the 3′ end	NR	catalytically inactive	∙ The solubilized fusion protein was used to raise antibody.	Suyama et al., 2002
PceA1/PceA2^a^	Environmental sample	*E. coli* BL21	His-taq	NR	catalytically inactive	∙ The enzyme synthesized *in vitro* exhibited PCE to TCE dechlorination activity at 2.3 μmole/h ml.	Kimoto et al., 2010
PceA	*D. restrictus*	*E. coli* BL21 (DE3)	Strep-Tag II	*E. coli* trigger factor	catalytically inactive	∙ TAT signal sequence was removed ∙ The recombinant PceA could bind methylcobalamin and the Fe–S clusters could be reconstituted chemically.	Sjuts et al., 2012
PceA	*D. hafniense* strain Y51	*Shimwellia blattae* (internally synthesize cobamides)	N-terminal Strep-tag II	PceT	8.33 (in crude extracts)	∙ The first functional recombinant RDase enzyme ∙ The presence of 5,6-DMB and hydroxocobalamin supported protein expression	Mac Nelly et al., 2014
NpRdhA	*Nitratireductor pacificus* pht-3B	*Bacillus megaterium* MS941 (internally synthesize cobamides)	C-terminal His-tag	NR	9.8 (3,5-dibromo-4-hydroxybenzoic acid)	∙ This cytoplasmic enzyme lacks both TAT signal and the RdhB subunit, and could be purified under aerobic conditions.	Payne et al., 2014
VcrA	*Dehalococcoides mccartyi* strain VS	*E. coli* BL21 (DE3)	N-terminal His-tag, Maltose Binding Protein (MBP)-tag, TEV protease cleavage site	NR	2.25	∙ TAT signal sequence was removed ∙ Hydroxocobalamin/adenosylcobalamin and Fe-S clusters in the presence of mercaptoethanol were reconstituted. ∙ Catalyzes dihaloelimination of 1,2-DCE to ethene	Parthasarathy et al., 2015

## Determination of reductive dehalogenase substrate specificity

Given that ORB harbor multiple reductive dehalogenase genes, the prediction of substrate specificity based on sequence information is difficult (Hug et al., 2013). Buttet et al discussed sequence-substrate relationships within their work and concluded that sequence similarity and substrate specificity are generally not correlated (Buttet et al., 2013). Although RDases contain highly conserved domains, the authors emphasized that even minimal variations in the sequence are responsible for different substrate ranges. Functional assays require the isolation and purification of the native biocatalyst and thereby remain a bottleneck to assess substrate specificity in many cases (Richardson, 2013).

**Figure 3:**
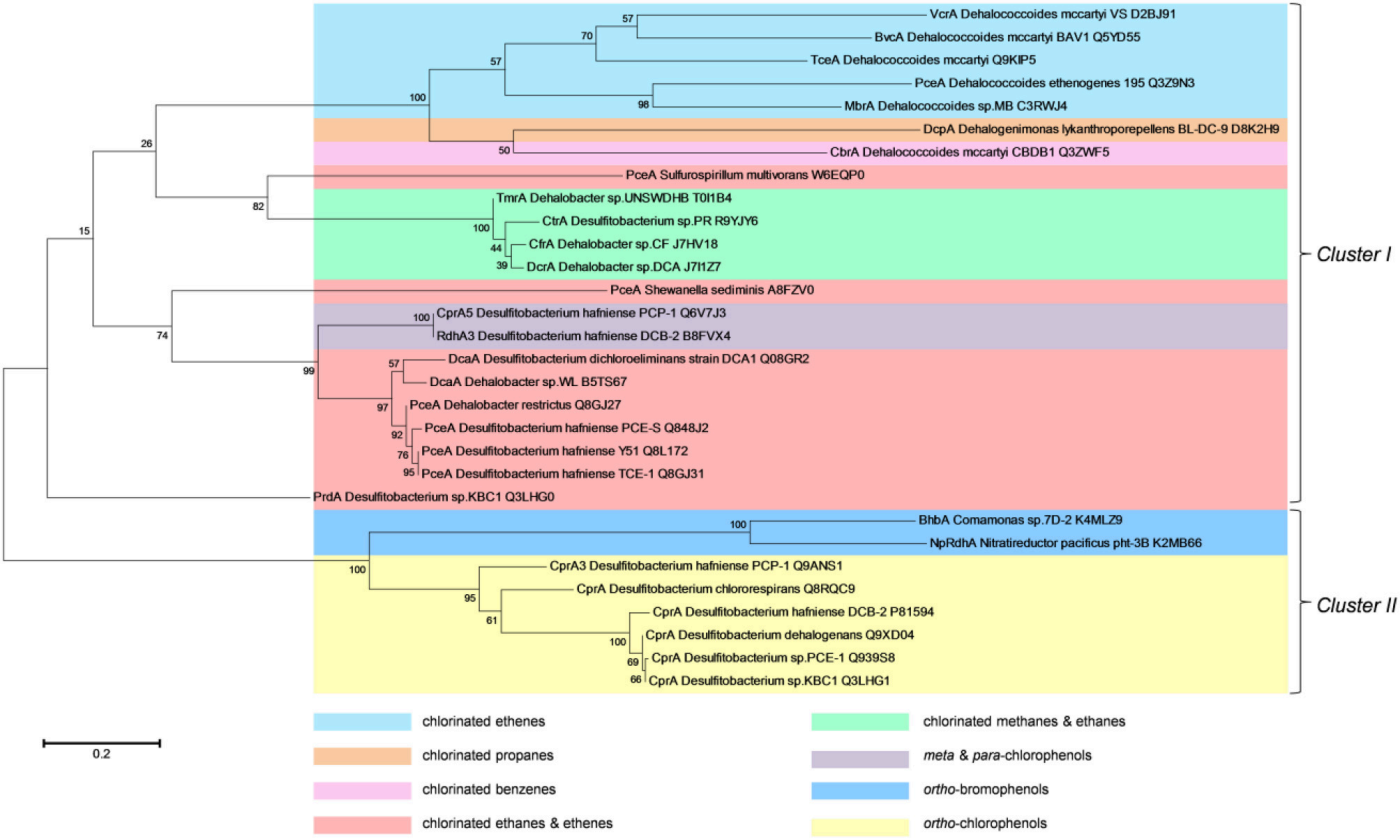
**Maximum Likelihood phylogenetic analysis of reductive dehalogenases characterized to date**. The evolutionary history was inferred by using the Maximum Likelihood method based on the JTT matrix-based model (Jones et al., 1992). The tree with the highest log likelihood (−7407.3363) is shown. The percentage of trees in which the associated taxa clustered together is shown next to the branches. Initial tree(s) for the heuristic search were obtained by applying the Neighbor-Joining method to a matrix of pairwise distances estimated using a JTT model. The tree is drawn to scale, with branch lengths measured in the number of substitutions per site. The analysis involved 30 amino acid sequences. All positions containing gaps and missing data were eliminated. There were a total of 201 positions in the final dataset. Evolutionary analyses were conducted in MEGA6 (Tamura et al., 2013).

Given these issues, we performed a maximum likelihood-based phylogenetic analysis based on the amino acid sequence of the only RDases characterized to date. This analysis reveals two main clusters with each having different clades arranged according to their substrate specificity (Figure 3). Most of these RDases are grouped within the first cluster (cluster I), with their functional similarity against certain substrates, such as chlorinated ethenes, chlorinated propanes, chlorinated benzenes, chlorinated ethanes and ethenes, chlorinated methanes and ethanes, and meta/para-chlorophenols. The RDases studied in all *Dehalococcoides* members are also included in this cluster and they form a separate clade, with the exception of CbrA from *D. mccartyi* CBDB1. All trichloromethane and trichloroethane-reductases (TmrA, CtrA, and CfrA), sharing high sequence homology, are also grouped into the same clade. The second main group (cluster II) contains aliphatic organohalide-reducing enzymes, such as *ortho*-bromophenol and *ortho*-chlorophenol reductases. Two main clades included here represent a distinct class of RDases typically identified in aerobic microbes (BhbA and NpRdhA) and all CprAs found in *Desulfitobacterium* species, both of which catalyze *ortho*-halogenated phenols. There are however, RDases with similar function, such as PceA from *S. multivorans*, PceA from *S. sediminis* and all other PceAs that are located in different branches. Another interesting observation is that RDases with different functionality, such as DcpA from *Dehalogenimonas lykanthroporepellens* BL-DC-9 and CbrA from *D. mccartyi* CBDB1, group into the same clade, it can be speculated that these enzymes share a similar substrate specificity that is yet unknown. We contend that this phylogenetic clustering based on RDase amino acid sequences is potentially a useful tool for predicting functional specificity of these enzymes and this information could also be exploited for further specific genetic tools to study homologous proteins as putative RDases.

A broad range of substrate specificity has been reported among ORB. Different substrates for the TceA reductive dehalogenase from *Dehalospirillum multivorans* were evaluated by offering different electron donors and acceptors to the native enzyme and the reductive dechlorination of chlorinated propenes in addition to chlorinated ethenes were reported (Neumann et al., 2002). The PceA of *Desulfitobacterium* sp. Y51 was found to dechlorinate not only PCE and TCE but also various chloroethanes, especially those with a high number of chloride substituents (Suyama et al., 2002). TceA from *Dehalococcoides ethenogene*s also catalyzes the dehalogenation of various three to five carbon atom containing haloalkanes and haloalkenes besides its more favored substrate TCE, albeit at lower rates (Magnuson et al., 2000).

## Evolutionary aspects of ORB and their RDases

Horizontal gene transfer (HGT) is a characteristic evolutionary mechanism that is widely reported among various genera of ORB (Liang et al., 2012). This is supported by the discovery of various mobile genetic elements, including insertion sequences (*IS*), transposases (TnpA), genomic islands (GIs), prophages, and recombinases, which have been revealed to be adjacent to the partial or complete set of the *pceABCT* cluster.

A composite circularized transposon, Tn-*Dha1*, that contains two identical *IS*s (*ISDha1)* surrounding the *pceABCT* gene cluster was identified in *D. hafniense* strain TCE1 (Maillard et al., 2005), but in the absence of PCE, Tn-*Dha1* was rapidly lost (Banerjee and Ragsdale, 2003). In recent genomic studies of *Dehalobacter* strains, transposition also appears to be an important horizontal transfer mechanism for the reductive dehalogenase genes (*rdh* genes). However, further validation of this mechanism is required (Tang et al., 2012; Deshpande et al., 2013; Kruse et al., 2013).

Comparative genomic analyses of *Dehalococcoides* sp. strains VS and BAV1 as well as other enrichment cultures (ANAS and KB-1) revealed horizontal transfer of the VC reductase encoding genes *vcrABC* and *bvcAB* that were embedded in GIs specifically integrated at the single-copy gene, *ssrA*, encoding a structural RNA (McMurdie et al., 2009, 2011). Also, microarray transcriptomic studies proved the up-regulation of bacteriophage and transposase genes was observed in *D. ethenogenes* strain 195 and a mixed microbial consortium (KB-1) in response to starvation (Grostern et al., 2010).

Interestingly, most of the *rdhAB* operons are not passengers of HGT through recently acquired GIs; instead, they are inherited vertically, as only a few of these operons are located in the GIs. Of a total of 19 putative *rdhAB* operons in *D. ethenogenes* 195 only three of them are found in GIs. Similarly, only the *cfrAB* operon (out of 17 *rdhAB* operons) in the *Dehalobacter* CF was found within a GI. For *Desulfitobacterium dehalogenans*, none of its six *rdhAB* operons are found in a GI. Nevertheless, it is obvious that ORB genomes are dynamic, harboring many GIs. Also, diversity is observable in *rdhAB* regions. One possible explanation for this is the evidence of various mobile genetic elements (MGEs) in the close proximity of *rdhAB* operons, and many *rdhAB* related genes (e.g., PceC, PceT, CRP etc.) are seriously disrupted by MGE insertion. In case of *Desulfitobacterium hafniense* Y51 and TCE-1, they also have catabolic transposons harboring *rdhAB* operons. This type of transposon is functional and easily travels among bacteria.

HGT is important for microorganisms exposed to environmental stress caused by toxic polychlorinated pollutants to develop acquired catabolic pathways from even phylogenetically distinct bacteria in order to adapt to contaminated ecosystems and compete in the microbial community. The cells equipped with reductive dehalogenation capacity via HGT of *rdh* genes from other ORB can become able to use organohalides either in their respiratory process as the terminal electron acceptors or co-metabolically to permit tolerance to high levels of organochlorines (Liang et al., 2012). This may also lead to formation of a large proportion of the microbial community that is best equipped for given organochlorine degradation and further facilitate a HGT-mediated *in situ* bioremediation approach.

## Role and significance of ORB and RDases in bioremediation

Anaerobic reductive dehalogenation can be a critical initial step in the bioremediation of many organohalides (for example, PCE/TCE to *cis*-DCE); however, complete detoxification usually requires further aerobic oxidation of the dechlorination products, such as *cis*-DCE and VC (Coleman et al., 2002). It should however, be noted that certain ORB-containing enrichment cultures, such as *Dehalococcoides*-containing KB-1 culture and *Dehalobacter*-rich AusCF culture, have been found to completely dechlorinate PCE to ethene (Major et al., 2002) and TCM to acetate and hydrogen (Lee et al., 2012), respectively. Although such complete reductive dehalogenation under anaerobic conditions is feasible, great attention should be paid to the risk associated with the accumulation of similarly toxic *cis*-DCE and VC, which may require further biostimulation with excess electron donors. Such additional stimulation can cause further competing processes such as nitrate and sulfate reduction and methanogenesis (Sun and Cupples, 2012; Révész et al., 2014). Hence, development of processes based on sequential stimulation of anaerobic-aerobic biodegradation has attracted significant attention and has been demonstrated (Thullner et al., 2012; Matturro et al., 2013) followed by further electro-bioremediation approaches (Rossetti et al., 2008; Matturro et al., 2012). Alternatively, the complete dechlorination of TCE to ethene of the *Dehalococcoides* mixed culture, which already had TceA and VcrA, was further enhanced by additional inoculation of *Dehalococcoides* strain BAV1, which contains BvcA enzyme for dehalogenation of *cis*-DCE and VC to ethene, using anaerobic biotrickling filters (Futagami et al., 2011).

Numerous technical aspects must be taken into account for subsurface bioremediation technologies. A critical step is site assessment and process monitoring based on data obtained from site samples using chemical (such as contaminant identification and characterization, electron acceptor alternatives, local nutrients, redox potential, pH, and the presence of potential inhibitors of reductive dehalogenation) and microbiological analyses (molecular biological tools for microbial community diversity and activity analyses). Recent rapid advances in molecular biological tools have had a profound effect on the understanding of bioremediation processes in the field biological remedial processes and the most widely used tool is qPCR for the 16S rRNA gene for bacterial identification (Smits et al., 2004; Chen et al., 2014). It was recently demonstrated that TaqMan chemistry (the fluorogenic 5′ nuclease) is recommended for enumeration of *Dehalococcoides* 16S rRNA biomarker genes over SYBR Green I detection chemistry, where nonspecific amplification observed in groundwater sample assessment (Hatt and Löffler, 2012). Moreover, qPCR targeted to *rdhA* genes using degenerate and gene-specific primers provides more accurate information with regard to characterization of dechlorination activity of the microbial community (Regeard et al., 2004; Lee et al., 2006; Ritalahti et al., 2006; Cupples, 2008). The applicability of qPCR in the near term has further been strengthened by its extension to mRNA-based qPCR, which offer more direct assessment of dechlorination activity. Furthermore, a microfluidics-based, nanoliter qPCR platform has recently been designed for the quantification of *rdh* gene repertoires and applied to on-site quantitative analysis of microbial diversity (Mayer-Blackwell et al., 2014). Among other tools reported are denaturing gradient gel electrophoresis (DGGE; Grostern and Edwards, 2006; Futagami et al., 2011), terminal restriction fragment length polymorphism (T-RFLP; Major et al., 2002), fluorescence *in situ* hybridization (FISH; Rossetti et al., 2008; Matturro et al., 2012, 2013), and DNA-stable isotope probing (SIP) or compound specific stable isotope analysis (CSIA; Sun and Cupples, 2012; Thullner et al., 2012; Révész et al., 2014). The long-term need for molecular biology tools for bioremediation research and implementation has to some degree been met by recent advances in the “omics” (Regeard et al., 2004; Smits et al., 2004; Ritalahti et al., 2006; Hatt and Löffler, 2012) and array approaches; however, their full scale on-site practicability is yet to be established. On-site monitoring of genetic biomarkers is desirable and this was recently demonstrated by the design of a hand-held device (termed Gene-Z), which allows real-time and DNA extraction-free loop mediated isothermal amplification using primers targeted at *Dehalococcoides* spp. specific *16S rRNA* and *vcrA* genes (Grostern and Edwards, 2006).

Biostimulation approaches can facilitate the implementation of a successful bioremedy via adjustment of pH and redox potential with addition of base or external reducing equivalents (Dybas et al., 2002). A critical issue for successful *in situ* anaerobic bioremediation is the delivery of organic substrates or donors with sufficient loading rates and uniform distribution into the subsurface. Failure to do this may potentially lead to accumulation of regulated intermediate degradation products, such as *cis*-DCE and VC. Lactate, molasses, vegetable, or emulsified vegetable oil, hydrogen release compound (HRC®), mulch and compost are common electron donors but variable approaches have to be designed for different substrates, as each have differing physio-chemical properties (Henry, 2010). Microbiological bottlenecks, in particular the absence or insufficient quantity of ORB with desired dechlorination activity, can also be addressed via bioaugmentation approaches. Notable pioneering efforts were made on PCE detoxification by *Dehalococcoides* augmentation (Major et al., 2002; Lee et al., 2006) that paved a way to more recent field scale practices (Ritalahti et al., 2005; Cupples, 2008; Schaefer et al., 2009; Justicia-Leon et al., 2014; Révész et al., 2014). Genetic engineering of environmental microorganisms for bioremediation purposes could be a considered option, however, such genetic modification processes are scientifically challenging and are likely to be problematic in terms of regulatory policies (Snow et al., 2005; de Lorenzo, 2009).

Cell-free enzymatic bioremediation has been an attractive alternative to bioaugmentation, owing to its several advantages, such as growth-rate independence, potentially faster reaction rates, targeted and predictable activity, greater physiochemical tolerances, readily biodegradable, potential for improvement or modification by protein engineering, fermentation-based large-scale production and no contamination with genetic material (Sutherland et al., 2004; Russell et al., 2011). Notwithstanding these advantages, significant limitations and challenges still remain to be addressed. In view of enzyme production and their characteristics, major hurdles remain, including obtaining and maintaining RDases in a catalytically active form with sufficient yield, reconstitution of corrinoid cofactors and Fe-S clusters under anaerobic conditions, physical tolerance against extreme environmental conditions with variable pH and temperature and inexpensive production costs for pure enzymes. Additionally, negative effects of the pollutants present on the enzyme efficiency, costly purification processes of the free enzymes and enzyme instability under harsh environmental conditions may practically restrict the use of free-enzyme bioremedients. Whilst immobilized enzymes on suitable carriers may be more feasible (Gianfreda and Rao, 2004), their application in the bioremediation of organohalide-polluted sites remains some way off.

## Concluding remarks and future research

Recent advances in optimizing growth conditions of ORB, understanding metabolic pathways via omics-based approaches, expressing functional enzymes in recombinant forms and elucidating protein crystal structures have significantly progressed understanding of the microbial mechanisms of dehalogenation. Nevertheless, generation of a sufficient quantity of biocatalysts with high activity remains as a significant challenge for developing further understanding of structure-function relationships. The recombinant approach, whilst promising, requires significant development to produce functional holoenzymes with the correct corrinoid cofactor and Fe-S clusters. However, recombinant strategies have begun to demonstrate specific activities which are comparable to purified native enzymes and recombinant expression provides future capacity for the enhancement of the enzyme activity, tuning substrate specificity, and improving enzyme stability. Both rational (protein engineering) and irrational (directed evolution) approaches can be employed to increase recombinant enzyme diversity. Clustering of known enzymes based on amino acid sequence revealed in this study, suggests that rational approaches to engineering, (for example substrate specificity), can be informed by this type of analysis. In addition, the further exploration of novel niches, harboring microorganisms with organohalide-respiring capability via reductive dehalogenation, is expected to continue and yield new insights. More effort should be devoted to metatranscriptomic studies of microbial consortia to identify novel candidates with potential organohalide biodegradation applications. Metagenomic mining for novel *rdhA* genes from environmental samples further provides potential to discover new dehalogenation genes from non-culturable members of these niche environments.

## Author contributions

BJ, HE, SB, ML, CM, and MM contributed to the conception and design of the work and to the acquisition, analysis and interpretation of the data. All authors contributed to the drafting of the manuscript and approved the final version to be published.

## Funding

This work was in part supported by an ARC Linkage grant (LP130100454). BJ is the recipient of a University of New South Wales postgraduate scholarship.

### Conflict of interest statement

The authors declare that the research was conducted in the absence of any commercial or financial relationships that could be construed as a potential conflict of interest.
